# Defining hospital markets – an application to the German hospital sector

**DOI:** 10.1186/s13561-014-0028-0

**Published:** 2014-11-26

**Authors:** Corinna Hentschker, Roman Mennicken, Andreas Schmid

**Affiliations:** RWI and Ruhr-Universität Bochum, Hohenzollernstr. 1-3, 45128 Essen, Germany; Landschaftsverband Rheinland, Kennedy-Ufer 2, 50679 Cologne, Germany; University of Bayreuth, JP Health Management, 95440 Bayreuth, Germany

**Keywords:** I11, K21, L11, L40, Hospital market, Concentration, Product market, Geographic market, Germany

## Abstract

The correct definition of the product market and of the geographic market is a prerequisite for assessing market structures in antitrust cases. For hospital markets, both dimensions are controversially discussed in the literature. Using data for the German hospital market we aim at elaborating the need for differentiating the product market and at investigating the effects of different thresholds for the delineation of the geographic market based on patient flows. Thereby we contribute to the scarce empirical evidence on the structure of the German hospital market. We find that the German hospital sector is highly concentrated, confirming the results of a singular prior study. Furthermore, using a very general product market definition such as “acute in-patient care” averages out severe discrepancies that become visible when concentration is considered on the level of individual diagnoses. In contrast, varying thresholds for the definition of the geographic market has only impact on the level of concentration, while the correlation remains high. Our results underline the need for more empirical research concerning the definition of the product market for hospital services.

## Background

Many hospital markets are undergoing structural changes. In those countries in which patient choice and competition between providers are fostered, merger control is a predominant and controversially discussed issue. Especially the adequacy of hospital market definitions is frequently disputed. These definitions are nontrivial, as both dimensions – the product and the geographic market – need to be correctly specified. The denied merger between two NHS Foundation Trust Hospitals in southern England [1] and the controversy around three approved merger cases in the Netherlands [2] are very recent examples. In Germany, questions around the correct definition of the product and geographic market have puzzled the antitrust authorities [3], government advisory bodies [4,5], courts [6] and economists [7] for many years. As we will show, consensus on the correct approach has not yet been achieved.

Over the past decade, the German hospital market has been under continuous transformation including considerable mergers and acquisitions (M&A) activity, affecting the structure of the hospital market, access to care and the power balance between hospitals and insurers considerably. Intensified by payment reforms and other factors – such as shrinking financial resources of municipalities owning public hospitals and a pro market attitude in health politics – hospitals’ self-conception has been transformed [8,9]^a^. They became – and to some extent were forced by these external factors to become – players in the health care market, built up management skills and started to make strategic decisions to improve their financial performance and their customer base. Thus, M&A activity could be observed both on a local level, forming hospital systems with a small number of hospitals, and on a supra-regional level, forming hospital chains that are active in various local hospital markets across the country. Although the German Antitrust Authority oversees M&A activity in the hospital sector, there are concerns that this may not prevent highly concentrated hospital markets that would allow to exercise market power [10]^b^.

Making use of data on the German hospital market, this paper addresses focal issues regarding the definition of the product market and the geographic market. Commonly, the product market definition assumes a cluster market, i.e. summarizing all hospital services under the label “acute in-patient care”. We discuss the limitations of this definition and provide evidence that important information is omitted by this approach thereby supporting the approach taken by the English antitrust authorities in the aforementioned case. We split up the product market using exemplary diagnoses, for which we only consider hospitals as competitors which offer treatment in the respective diagnoses. Furthermore, the size of geographic hospital markets is frequently driven by arbitrary thresholds. We test the robustness of our results over a range of different threshold combinations.

This study contributes to the existing literature in the following ways. Firstly, the analysis provides empirical evidence on the effects of varying product market definitions on the level of measured concentration, emphasizing the urgent need for more empirical research on this issue [11,12]. Secondly, the robustness of definitions of the geographic market is tested over a number of threshold combinations. Thirdly, this is only the second comprehensive analysis of the German hospital sector that accounts for hospitals’ system membership, an often neglected but important issue as hospitals owned by the same entity do not compete with each other.

In the following, we provide a review of concepts, as various methodological issues regarding the definition of the relevant market and their relevance for the situation in Germany have not yet been sufficiently explored. In the next section, we describe the dataset and compare it to the dataset of the Federal Statistical Office. Furthermore, we discuss our approach to define the relevant product and geographic market and explain the calculation of the indicators for market concentration. Thereafter we present the results, followed by a discussion of the implications and limitations of this study in the last section.

## Review of concepts

We identify three strands of literature that are relevant to our work: studies on the definition of the product market, literature on the definition of the relevant geographic market and analyses of the dynamics and the structure of the German hospital market. We will not cover general theoretical or empirical studies on hospital markets as these primarily refer to the U.S. and have already been extensively reviewed elsewhere [12-16].

If the relevant market is adequately defined, it includes all relevant substitutes in the product as well as in the geographic dimension. The most common tests for both dimensions rely on the analysis of marginal price changes and their effect on demand [17]. In hospital markets, however, most patients are covered by health insurance and thus less susceptible to any changes in prices. Furthermore, prices in the health sector are rarely outcomes of market processes but are set or at least controlled by regulatory entities. This is also true for the German hospital market; patients are fully insured by the Statutory Health Insurance and prices are set by a system based on DRGs reflecting average costs. Thus, alternative approaches are required.

### Definition of the product market

Besides the stance that there is one general market (cluster market) for acute inpatient care, there are three options to differentiate product markets that are commonly discussed [7]:^c.^ Firstly, product markets can be separated by care level of the hospital, i.e. a differentiation between hospitals offering basic services, intermediate services and highly complex specialized services. Opponents argue that this is not feasible as there is too much overlap between these categories. Secondly, it is possible to distinguish between specialty departments. However, this separation is by no means binding. In many cases department structures just reflect organizational deliberations, while the allocation of specific conditions or diagnoses can vary between hospitals. Thirdly, each diagnosis can potentially be seen as a separate product, as patients seek care for a specific condition and cannot substitute this care by treatment for another condition. The latter is typically criticized for being far too narrow and not reflecting reality.

Inappropriate market definitions may result in misguided decisions by antitrust authorities and biased research results. Following Zwanziger et al. we argue that many hospitals do not compete for generic acute care patients, but e.g. for orthopedic or cardiac patients [11]. This is due to various reasons, such as that they simply do not offer the other service or that their profit margin or level of expertise is higher for one than for the other. As Lindrooth illustrates, summarizing all types of hospital services under one product market definition may create misleading depictions of the reality [12].

Zwanziger et al. favor an approach that considers supply-side substitutability, i.e. the ability of hospitals to employ physicians and facilities for different groups of diagnoses or procedures [11]^d^. Varkevisser et al. sugest a similar approach for the Dutch setting [18]. In a practical application, the Office of Fair Trading and the Competition Commission have analyzed the merger of two NHS Hospital Foundation Trusts by differentiating more than thirty (sub-)specialties as well as separating elective, emergency and out-patient services. Extensive primary data collection was conducted, including in-depth interviews with all involved stakeholders to find a definition of the product market that fits this very specific case [1]. However, to this date, this method has not been evaluated in more detail and warrants more research before a potential application to the German hospital market can be considered^e^.

Thus, the dominating and rarely challenged approach in merger control practice as well as in research is the cluster market, i.e. some sort of “general acute care hospital services” (p. 1423) [13]. This is true for the U.S. and for the Netherlands but to some extent also for Germany, where the Antitrust Authority for various reasons is still reluctant regarding a considerable disaggregation of the product market [19].

### Definition of the geographic market

Concerning the geographic dimension, some analyzes rely on geopolitical boundaries. However, it is obvious that these boundaries do not necessarily match with real hospital markets. Furthermore, two hospitals just next to each other but on opposite sides of a district border would be classified as not competing against each other. The analysis of patient-flow data has proven to mitigate some of these problems and to be a pragmatic and reliable – while by no means perfect – approach. By looking at patient flows, one can either aim at identifying rather self-contained areas (e.g. following the Elzinga-Hogarty approach^f^) or at identifying the relevant catchment/distribution area. In the latter case, the analysis usually starts with a very small geographic area which is step by step enlarged until the marginal increase of patients that patronize the respective hospital is below a certain threshold or a sufficiently large share of the patients treated by the hospital are covered. To do so either circles with incrementally increasing radii can be drawn around a hospital or small geographic units like ZIP code areas can be successively added. Using small geographic units is superior to circular methods as this approach allows for more flexible markets that align to real infrastructure and settlement patterns. However, the decisions on the levels of these thresholds cannot be backed up by sound theoretical arguments. This implies that at least some sensitivity analyses are required to evaluate the robustness of the results when thresholds are varied. These approaches as well as variations thereof are explained, analyzed and discussed in more detail in studies such as [11,20,21].

Recent approaches based on hospital choice models are much more demanding on the data available and are prone to criticism such as strong assumptions regarding the direct proportionality between price and time elasticities [12,18,21]. To discuss or to implement such a method is beyond the scope of the current study. However, compared to such structural models, figures on market concentration based on patient flows usually provide rather conservative estimates, i.e. they underestimate the true market concentration [22].

Summing up, for both product market and geographic market definition the first best approach can hardly be implemented in the hospital sector. Although some “common practice” has emerged over time, there is no consensual gold standard for either dimension of market definition. The core consensus remains, that more theoretical and empirical research is required. Turning now towards the available evidence regarding the German hospital sector this need is even more emphasized.

### The German hospital sector

In 2011, the expenditures for hospital services in Germany totaled 77 billion euros or 26% of all health expenditures. The capacity in terms of hospital beds has been fairly constant since 2009 at around 502,000 beds. In the past years a continuous increase of in-patient cases is observable, i.e. between 2005 and 2011 the number of in-patients increased by 11% from 16.5 to 18.3 million. This goes along with a decreasing length of stay: The average length of stay was around 7.7 days in 2011 compared to 8.7 days in 2005. The complementary public funding on the basis of the Hospital Financing Act has been declining for years, which has caused the cumulative investment gap across all German hospitals to grow to an estimated 15 billion euros. For many providers severe economic difficulties are the consequence. This may be one reason for the ongoing M&A activities that change the structure of hospital markets. The number of hospitals is decreasing, while the number of hospitals which organize themselves in hospital systems increases continuously [23].

In the context of these developments, concentration in the German hospital sector is a topic being discussed – although not excessively – in the pertaining legal and economic literature, the record of decisions of the German Antitrust Authority usually being the focal point of the analyzes. However, neither courts and legal experts (e.g. [19] or [24]) nor economists (e.g. [7] or [25]) have yet reached a consensus on an appropriate product market definition. The literature conveys the impression that most experts feel that the cluster market approach applied by the German Antitrust Authority may result in an incorrect depiction of the true competitiveness of the market. At the same time it is not clear, if this is really the case and which alternative approach would be more suitable^g^. Most arguments are supported by fictive scenarios that support or respectively discourage the use of one method over the other. Regarding the geographic dimension of the market definition, the approach of the German Antitrust Authority results in rather narrow markets, which most likely reflect the regional nature of hospital markets [25]. However, at the same time there is evidence that depending on the type of the treatment needed, patients are willing to travel much longer distances for some procedures than for others (see also the findings presented in the results section.

## Data and methods

One explanation for this ambiguity may be that there is only scarce empirical evidence on the structure of the German hospital market, because none of the available datasets contains a unique identifier for the owner of the individual hospitals. Most authors refrain from creating a hospital system identifier, but calculate concentration measures based on individual hospitals as a proxy for true market power^h^. Hence, it is inherently assumed that hospitals owned by the same entity behave like competitors. As this is a very strong assumption, we use the study by Schmid and Ulrich (SU) as a benchmark to our analysis [26]^i^. This study was the first to look at the structure of the German hospital market systematically accounting for individual hospitals’ system membership. The results of SU indicate that the German hospital market was highly concentrated in 2007. Any attempt of measuring market concentration on hospital level and thereby disregarding system membership significantly underestimates the level of concentration and potentially severely biases the results.

We use administrative data according to §21 KHEntgG (hospital remuneration law) generated by the German hospital payment system based on diagnosis related groups (DRGs)^j^. The dataset contains each in-patient treatment episode in Germany of the year 2007. Patient characteristics such as age, gender, main and secondary diagnoses, procedure codes as well as the ZIP code of residence are available. Furthermore, the dataset covers information on hospitals like ownership type, bed size, and teaching status. We exclude hospitals with less than 50 beds. In most cases these hospitals represent small specialist hospitals (e.g. orthodontics, plastic surgery, ophthalmology) which are of negligible relevance for the regular provision of hospital services. To this dataset we merge the hospital system identifiers as used by SU.

On the patient level we exclude accompanying persons, patients without a coded main diagnosis, patients with a missing or invalid ZIP code, and patients with a psychiatric condition as main diagnosis. With these exclusion restrictions applied, our full dataset (TOTAL) comprises a total of 16.6 million patients treated in 1,517 hospitals or rather 910 hospital systems (see Table 1). The share of single hospitals, i.e. hospitals which do not belong to a hospital system, is 45%. For all calculations based on the full dataset we use a 10% sample stratified on hospital and ZIP code levels. Robustness checks have shown that there are no significant differences between the results based on the 10% sample and the full dataset.

**Table 1:** **Descriptive statistics of the full sample and on diagnosis based sub-samples**

	**Number of patients**	**Number of hospitals**	**Number of hospital systems**	**Share of single hospitals (%)**	**Size of a hospital system (mean)**	**Size of a hospital system (SD)**
**Total**	**16,561,426**	**1,517**	**910**	**45.0**	**1.67**	**2.25**
***Standard procedures***						
*Nonsurgical procedures*						
PNEU**	200,618	1,294	759	42.4	1.70	2.11
STROKE** ^1)^	274,743	1,287	762	42.9	1.69	2.04
BIRTH	604,436	860	579	52.0	1.49	1.56
*Surgical procedures*						
APP**	68,198	1,108	679	45.8	1.63	1.96
CHOL*	154,667	1,147	687	44.3	1.67	2.08
*Orthopaedic surgical procedures*						
HAP	138,102	1,016	654	49.5	1.55	1.90
KAP	135,236	935	617	51.3	1.52	1.77
HIP***	98,084	1,118	680	45.0	1.64	2.05
ENDO	371,422	1,207	728	44.9	1.66	2.16
***Complex surgical procedures***						
AAA	8,210	386	305	69.9	1.27	1.07
CABG ^2)^	35,916	76	62	69.7	1.23	0.73

Notes: For diagnoses market with */**/*** more than 25%/50%/75% of all admissions were emergency admissions; ^1)^13% of all stroke patients were transferred from another hospital; ^2)^32% of all CABG patients were transferred from another hospital. For all other diagnoses the share of transferred patients was less than 5%. PNEU - pneumonia; APP - appendectomy; CHOL - cholecystectomy; HAP - hip arthroplasty; KAP - knee arthroplasty; HIP - hip fracture; ORTHO - joint category for HAP, KAP and HIP; AAA - intact abdominal aortic aneurysm; CABG - coronary artery bypass surgery.

As outlined in more detail below, when investigating potential alternative definitions of the product market, we create new datasets that only include patients with certain diagnoses. These datasets are much smaller than the original dataset, so we can refrain from drawing a subsample but use all observations available. To ensure data quality, we apply further restrictions on these datasets: Patients who are younger than 20 years are excluded for all diagnoses but BIRTH. Following the definition of Mansky et al., we exclude patients younger than 7 and older than 59 years for BIRTH [27]. In all diagnoses, except AAA, we exclude hospitals with less than ten cases. Because of the lower prevalence we exclude hospitals with less than three cases in the provision of care for AAA patients. The notion behind this approach is that these hospitals are of negligible relevance for the provision of the respective hospital services. They may need to treat those patients rather unexpectedly in case of emergency.

As expected, standard procedures are more frequent than complex surgical procedures. The number of hospitals offering treatment for certain diagnoses varies considerably (see Table 1). While almost all hospitals offer services for PNEU, only 76 hospitals in our sample offer CABG. The share of single hospitals does not differ markedly between the conditions except for AAA and CABG, where still 70% of all hospitals which treat patients in these conditions are stand-alone hospitals. At the same time, AAA and CABG are rather rare and complex conditions. It is plausible that only few highly specialized tertiary care hospitals provide these services and that within a hospital system the service is likely to be concentrated in one facility.

To calculate concentration measures, we first have to define the product and geographic dimensions of the relevant market. As basis for the product market we use all German hospitals which offer “acute in-patient care” as our benchmark. It is beyond the scope of this paper to develop a full model for a differentiated product market along the lines of Zwanziger et al. or Varkevisser et al. [11,18]. However, we want to explore if more research into this topic is advised. To do so, we test if a differentiation of the product market creates results that reflect the results of the standard product market definition, since severe discrepancies between the results would indicate that the use of an aggregated measure is not appropriate.

To decompose the product market we identify ten diagnoses that represent a wide range of hospital admissions, covering nonsurgical and surgical procedures, standard and complex as well as elective and emergency cases; i.e. pneumonia (PNEU), stroke (STROKE), birth (BIRTH), appendectomy (APP), cholecystectomy (CHOL), hip arthroplasty (HAP), knee arthroplasty (KAP), hip fracture (HIP), intact abdominal aortic aneurysm (AAA) and coronary artery bypass surgery (CABG). To capture the notion of supply substitutability, i.e. providers can easily rededicate staff and other resources between different types of treatment, we also form one joint category ORTHO for the three orthopedic treatments HAP, KAP and HIP (see Table 2)^k^.

**Table 2:** **Inclusion and exclusion restrictions for the conditions**

**Condition**	**Incl./Excl.**	**Diagnosis codes**	**Procedure codes**
PNEU	Incl.	A48.1; J10.0; J11.0; J12.*; J13; J14; J15.*; J16.*; J17.*; J18.*	
	Excl.	E84.*	
STROKE	Incl.	I60.*; I61.*; I63.*; I64	
BIRTH	Incl.	O00.* - O99.*	5-720.*; 5–724; 5–725.*; 5–727.*; 5–728.*; 5–729; 5–730; 5–731; 5–732.*; 5–733.*; 5–738.*; 5–739.*; 5–740.*; 5–741.*; 5–742.*; 5–745.*; 5–749.*; 9–260; 9–261; 9-268
APP	Incl.	K35.*; K36; K37	5-470.*
CHOL	Incl.	K80.*; K81.*	5-511.0*; 5–511.1*; 5–511.2*; 5–511.x; 5–511.y
	Excl.	C*	5-511.3; 5–511.4*; 5–511.5*
HAP	Incl.	M16.*	5-820.0*; 5–820.2*; 5–820.3*; 5–820.4*; 5–820.x*; 5–820.8*
	Excl.	M84.15; M96.0; S32.4; S72.0*; S72.1*; S72.2; T84.1	
KAP	Incl.	M17.*	5-822.1*; 5–822.2*; 5–822.3*; 5–822.4*; 5–822.6*; 5–822.7*; 5–822.9*; 5–822.a*; 5–822.b*
HIP	Incl.	S72.0*; S72.1*	5-790.*e; 5–790.*f; 5–793.*e; 5–793.*f; 5–794.*e; 5–794.*f; 5-820.0*; 5–820.2*; 5–820.3*; 5–820.4*; 5–820.x*; 5–820.8*
AAA	Incl.	I71.4; I71.02	5-384.5; 5–384.6; 5–384.7; 5-38a.1
CABG	Incl.	I20.*; I25.*	5-360.*; 5–361.*; 5–362.*; 5–363.*; 5–369.*
	Excl.	I21.*; I22.*	5-350.*; 5–351.*; 5–352.*; 5–353.*; 5–354.*; 5–355.*; 5–356.*; 5–357.*; 5–358.*; 5-35a.*; 5–359.*; 5–370.*; 5–371.*; 5–372.*; 5–373.*; 5–374.*; 5–375.*

Notes: The included diagnosis code is always related to the coded main diagnosis. The exclusion restriction for the diagnosis code is related to the secondary diagnosis. If diagnosis code and procedure codes are specified, a patient is only included in the sample if in each category at least one code exists. Diagnosis codes are based on the international statistical classification of diseases (ICD-10-GM). Procedure codes are based on the German classification system for procedures.

PNEU - pneumonia; APP - appendectomy; CHOL - cholecystectomy; HAP - hip arthroplasty; KAP - knee arthroplasty; HIP - hip fracture; AAA - intact abdominal aortic aneurysm; CABG - coronary artery bypass surgery.

Turning towards the geographic market, every hospital system has a specific catchment area from which most of its patients come from. This area is the relevant market, which is unique for every hospital system (HS). The procedure for defining the geographic market is based on the cumulative-marginal rule used by SU^l^. The approach analyzes patient flows on (five digit) ZIP code level. In the first step, the cumulative-threshold is relevant. The relevant market of a HS consists of the minimal number of ZIP code areas needed to account for x% of all patients treated by the HS (cumulative-threshold value). To get this, from a HS point of view all ZIP code areas are sorted in descending order according to the number of the treated patients in the respective HS. Starting with the first ZIP code area (with the highest number of patients), every ZIP code is added to the market until the cumulative number of patients within the added ZIP code areas reach a defined threshold. For example, with a threshold of 60% the procedure stops, if the patients within the assembled total ZIP code area cover 60% of the HS’s patient volume. In the second step, the marginal threshold is considered. Additionally to the ZIP codes that are identified by the cumulative threshold, all ZIP code areas are added to the market that account for at least y% of all patients treated by the HS (marginal threshold). The reasoning behind this threshold is that these areas are also important for the hospital system as a sufficiently large number of patients come from these ZIP codes. For example, if this threshold is set at 1%, every ZIP code that is not yet covered under the cumulative threshold but exceeds the critical number 1% of the HS’s patients is also added to the relevant market. The result is the 60/01-rule, which serves as our benchmark. As by definition such thresholds are always arbitrary, we also look at variations of the cumulative threshold at 40% and 80% and use 3% and 5% as alternative marginal thresholds.

Subsequently, we calculate the market share for the considered hospital system and its competitors. The market share is defined as the number of a hospital system’s patients in the relevant market divided by the total number of patients in the relevant market. This results in the Herfindahl Hirschman Index (HHI) which is defined as the sum of all squared market shares of all competitors in the market. The HHI is a measure for assessing market concentration and can range from 0 to 1: Values close to zero indicate that a HS operates in a market with high competition and low concentration; values higher than 0.18 indicate that a HS operates in a market with less competition and high concentration [13]^m^.

Other proxies of market concentration are the market share (MS), the number of competitors (NC), the cumulative market share of the three (CR3) largest competitors in the market and the number of ZIP codes (NZIP) that a market comprises^n^. For NC and – although rarely binding – also for CR3 relevant competitors have to be identified. In the study by SU a competitor is deemed to be a relevant competitor when the respective hospital system treats more than 3% of its patients in at least one ZIP code area of the relevant market. Robustness checks with different thresholds and alternative definitions are also conducted.

SU also apply this relevant competitor restriction when calculating the HHI and the CR3. This means that the squared market shares of potential competitors that are not deemed relevant are not added to the HHI. This contradicts the logic of the HHI, which takes into account the limited significance of small competitors with small market shares. For this reason, we deviate from SU in this point in all of our calculations but the direct comparison of results with the benchmark study at the end of the results section. Thus, unless otherwise stated, we calculate all HHI and CR3 values including all hospitals as potential competitors.

In the current analysis we calculate all concentration measures on the level of the hospital system (HS). However, if we calculated the mean and the other statistical measures of these concentration measures on the basis of these 910 hospital systems, the results would be distorted as the HHI of a large hospital system would have the same weight as e.g. a small stand-alone 60 beds hospital. To avoid this, we follow SU and calculate all these measures on the basis of all 1,517 hospitals, i.e. we weight all measures with the number of hospitals per hospital system. All hospitals that belong to the same hospital system enter the calculation with the identical HHI that has been calculated on hospital system level. By doing so, we give greater weight to larger hospital systems^o^.

## Results

### General findings

Calculating the HHI based on the cluster market approach, we obtain a fairly high average HHI of 0.19 (see Table 3). With a HHI of 0.17, the median is just slightly lower. Looking at the averages disguises the fact that a considerable number of hospitals, i.e. more than 25% (HHI^p75^) of all hospitals, operate in highly concentrated markets, even if the higher threshold of the revised U.S. Federal Merger Guidelines are applied. The high concentration is also reflected both in the market shares (MS) of the hospital systems in their relevant market and in the corresponding concentration ratio (CR3). The average market share is 27% which means that on average a hospital system treats more than one fourth of all patients in its relevant market. 25% of all hospital systems have already a market share of at least 42% (MS^p75^). The concentration ratio (CR3) is on average 59%, meaning that on average three hospital systems treat more than half of the patients. Hospitals are on average confronted with 14 competitors (NC) with a median of 9 competitors in their relevant market, which on average consist of 35 ZIP codes (NZIP) with a median of 24 ZIP codes. Overall, the results of our analysis indicate that large parts of the German hospital sector can be characterized by very high levels of concentration.

**Table 3:** **Concentration for cluster market**

	**N**	**Mean**	**p1**	**p5**	**p25**	**p50**	**p75**	**p95**	**p99**
HHI	1517	0.19	0.04	0.06	0.12	0.17	0.26	0.39	0.55
MS	1517	0.27	0.00	0.01	0.12	0.26	0.42	0.59	0.73
CR3	1517	0.59	0.21	0.33	0.50	0.58	0.70	0.81	0.90
NC	1517	14.19	2	3	6	9	17	43	62
NZIP	1517	35.27	6	9	17	24	37	118	140

**Table 4:** **Measures of concentration for different product market definitions based on hospital system (mean)**

	**HHI**	**MS**	**CR3**	**NC**	**NZIP**	**Distance** ^**1)**^	**# of HS**
**Total**	**0.19**	**0.27**	**0.59**	**14.19**	**35.27**	**20.38**	**910**
***Standard procedures***							
*Nonsurgical procedures*							
PNEU	0.34	0.46	0.74	8.41	23.53	12.23	759
STROKE	0.30	0.38	0.71	8.47	24.84	16.78	762
BIRTH	0.31	0.45	0.76	5.92	26.36	12.92	579
*Surgical procedures*							
APP	0.33	0.47	0.72	8.73	23.75	15.19	679
CHOL	0.34	0.48	0.75	8.02	24.73	12.02	687
*Orthopaedic surgical procedures*							
HAP	0.19	0.30	0.59	13.60	38.12	25.58	654
KAP	0.19	0.31	0.59	13.09	38.69	23.80	617
HIP	0.42	0.55	0.80	6.29	21.39	11.22	680
ORTHO	0.20	0.32	0.61	12.10	36.91	21.10	728
***Complex surgical procedures***							
AAA	0.44	0.60	0.85	6.11	21.97	26.51	305
CABG	0.56	0.70	0.92	1.58	61.96	50.97	62

Notes: ^1)^Data was censored at the 99th percentile to eliminate extreme outliers. PNEU - pneumonia; APP - appendectomy; CHOL - cholecystectomy; HAP - hip arthroplasty; KAP - knee arthroplasty; HIP - hip fracture; ORTHO - joint category for HAP, KAP and HIP; AAA - intact abdominal aortic aneurysm; CABG - coronary artery bypass surgery.

In the current analysis we calculate all concentration measures on the level of the hospital system (HS). Due to the lack of appropriate data, existing studies perform their analysis usually on the hospital level (H) (one exception is the study of SU). Comparing the results of both approaches, average concentration is lower when measured on hospital level, i.e. the average HHI (0.19 vs. 0.15), the average market share (0.27 vs. 0.18), and the average number of ZIP codes (35.27 vs. 22.19) decrease while the average number of competitors and the average CR3 stay roughly constant.

Although these differences are considerable, they still disguise the full extent of the distortion. Most importantly, the deviations average out. This is supported by the average absolute difference between the HHI calculated on hospital system level (HHI^HS^) and the HHI on hospital level (HHI^H^). The mean difference of these two measures is 0.06, which is considerably higher than the respective difference of the means, indicating that the difference of the means is not able to capture this discrepancy^p^. When looking at the correlation between the HHI^HS^ and the HHI^H^, we see a correlation of 0.58. This is a low correlation considering that HHI^HS^ and HHI^H^ are frequently used as if they were interchangeable measures.

**Figure 1:**
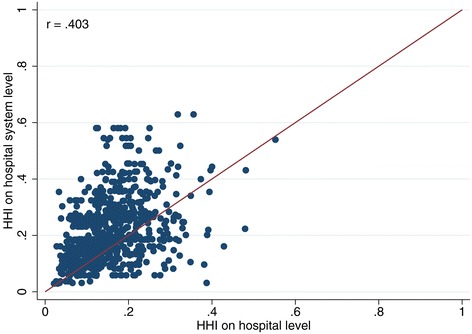
**Comparison of HHI based on hospital system level and on hospital level (only hospitals that are part of a hospital system).**

**Figure 2:**
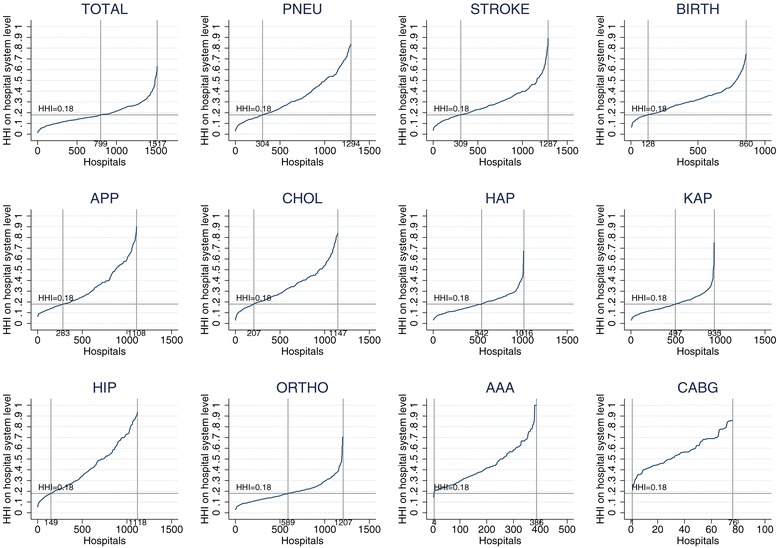
**Comparison of HHI based on hospital system level and on hospital level (stand-alone hospitals only).**

**Figure 3:**
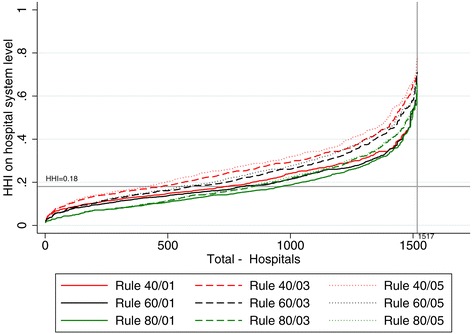
**Comparison of HHI based on hospital system level and on hospital level (all hospitals).**

Furthermore, in 2007, only about 45% of all hospitals were still stand-alone hospitals and this figure is steadily decreasing over time. This means that – comparing the HHI^HS^ with the HHI^H^ only for these stand-alone hospitals – the HHI is only different, if two or more competitors belong to the same hospital system^q^. The smaller the number of stand-alone hospitals becomes, the worse is the quality of HHI^H^ as a proxy for HHI^HS^. If only hospitals that belong to a hospital system are considered, the correlation drops to 0.403. The scatter plot in Figure 1 shows the latter scenario and provides a graphic illustration for the low correlation. For contrast, also see Figure 2 (only stand‐alone hospitals without system membership, r = 0.88) and Figure 3 (all hospitals combined, r = 0.58).

Summarizing, we argue that although used as a standard measure in econometric studies on the German hospital market, the HHI^H^ is no good approximation of true market concentration and that the HHI^HS^ should be used instead.

### Product market definition

We now disaggregate the product market, looking into a number of individual diagnoses. Table 4 shows the average concentration measure for every condition (see Table 5 for comprehensive descriptive statistics). Looking at the HHI it appears that only for HAP and KAP the HHI is in a similar range as the HHI of the total market. For all other diagnoses, the concentration is considerably higher, ranging from 0.30 for STROKE to 0.56 for CABG. For most diagnoses the HHI is just above 0.3. When testing for differences between HHIs for each diagnosis we have to reject the null hypothesis that the means are equal. The correlations between the HHI calculated on the basis of various subsamples are very heterogeneous. While some diagnoses seem to be closely related, others differ considerably (see Table 6).

**Table 5:** **Concentration measures for single conditions**

**Condition**		**N**	**Mean**	**p1**	**p5**	**p25**	**p50**	**p75**	**p95**	**p99**
AAA	HHI	386	0.44	0.20	0.21	0.29	0.40	0.57	0.78	1.00
AAA	MS	386	0.60	0.19	0.33	0.45	0.58	0.75	0.88	1.00
AAA	CR3	386	0.85	0.56	0.60	0.76	0.86	0.94	1.00	1.00
AAA	NC	386	6.11	1	1	2	4	8	19	30
AAA	NZIP	386	21.97	2	3	7	14	35	59	79
APP	HHI	1,108	0.33	0.09	0.11	0.18	0.27	0.46	0.68	0.81
APP	MS	1,108	0.47	0.09	0.16	0.29	0.45	0.65	0.82	0.90
APP	CR3	1,108	0.72	0.41	0.47	0.62	0.73	0.84	0.94	0.97
APP	NC	1,108	8.73	1	2	5	8	11.5	21	26
APP	NZIP	1,108	23.75	5	8	14	21	29	52	77
BIRTH	HHI	860	0.31	0.09	0.14	0.21	0.30	0.38	0.57	0.69
BIRTH	MS	860	0.45	0.07	0.17	0.32	0.45	0.59	0.73	0.83
BIRTH	CR3	860	0.76	0.39	0.54	0.68	0.78	0.85	0.94	0.97
BIRTH	NC	860	5.92	1	2	3	5	7	15	18
BIRTH	NZIP	860	26.36	6	9	15	23	29	73	96
CABG	HHI	76	0.56	0.25	0.32	0.44	0.56	0.69	0.85	0.86
CABG	MS	76	0.70	0.13	0.37	0.61	0.73	0.83	0.92	0.92
CABG	CR3	76	0.92	0.73	0.80	0.90	0.92	0.96	0.99	1.00
CABG	NC	76	1.58	1	1	1	1	2	3	4
CABG	NZIP	76	61.96	21	27	36.5	47	68	154	154
CHOL	HHI	1,147	0.34	0.07	0.11	0.21	0.32	0.44	0.68	0.82
CHOL	MS	1,147	0.48	0.05	0.12	0.31	0.49	0.65	0.82	0.90
CHOL	CR3	1,147	0.75	0.34	0.48	0.67	0.75	0.88	0.94	0.97
CHOL	NC	1,147	8.02	1	2	4	6	9	28	36
CHOL	NZIP	1,147	24.73	5	8	14	22	29	59	79
ENDO	HHI	1,207	0.20	0.05	0.08	0.13	0.18	0.24	0.38	0.62
ENDO	MS	1,207	0.32	0.03	0.06	0.19	0.30	0.44	0.59	0.72
ENDO	CR3	1,207	0.61	0.28	0.35	0.50	0.60	0.72	0.86	0.93
ENDO	NC	1,207	12.10	1	2	4	7	13	52	77
ENDO	NZIP	1,207	36.91	5	9	16	23	32	164	240
HAP	HHI	1,016	0.19	0.04	0.07	0.12	0.18	0.24	0.37	0.46
HAP	MS	1,016	0.30	0.04	0.06	0.17	0.28	0.43	0.55	0.64
HAP	CR3	1,016	0.59	0.26	0.37	0.49	0.59	0.70	0.83	0.90
HAP	NC	1,016	13.60	2	3	6	9	15	53	75
HAP	NZIP	1,016	38.12	5	8	18	26	35	148	245
HIP	HHI	1,118	0.42	0.09	0.13	0.23	0.38	0.57	0.84	0.91
HIP	MS	1,118	0.55	0.08	0.16	0.35	0.56	0.74	0.91	0.95
HIP	CR3	1,118	0.80	0.42	0.53	0.72	0.83	0.92	0.97	0.98
HIP	NC	1,118	6.29	1	1	3	5	8	15	20
HIP	NZIP	1,118	21.39	4	7	12	18	26	51	70
KAP	HHI	935	0.19	0.04	0.07	0.12	0.17	0.24	0.34	0.54
KAP	MS	935	0.31	0.04	0.06	0.18	0.30	0.43	0.55	0.72
KAP	CR3	935	0.59	0.25	0.36	0.48	0.59	0.70	0.83	0.90
KAP	NC	935	13.09	2	3	6	8	14	51	70
KAP	NZIP	935	38.69	7	11	19	26	35	154	223
PNEU	HHI	1,294	0.34	0.06	0.10	0.19	0.31	0.49	0.70	0.82
PNEU	MS	1,294	0.46	0.03	0.08	0.24	0.46	0.67	0.83	0.90
PNEU	CR3	1,294	0.74	0.30	0.45	0.64	0.77	0.87	0.94	0.98
PNEU	NC	1,294	8.41	1	2	4	6	11	22	36
PNEU	NZIP	1,294	23.53	5	8	14	20	27	60	81
STROKE	HHI	1,287	0.30	0.07	0.10	0.18	0.27	0.38	0.57	0.79
STROKE	MS	1,287	0.38	0.02	0.05	0.17	0.37	0.56	0.74	0.89
STROKE	CR3	1,287	0.71	0.35	0.44	0.62	0.72	0.82	0.91	0.96
STROKE	NC	1,287	8.47	1	2	4	7	11	20	28
STROKE	NZIP	1,287	24.84	4	7	14	21	29	78	81

**Table 6:** **Correlation coefficients of HHI of different conditions**

**Condition**	**AAA**	**APP**	**BIRTH**	**CABG**	**CHOL**	**ENDO**	**HAP**	**HIP**	**KAP**	**PNEU**	**STROKE**	**TOTAL**
AAA	1.00											
APP	0.39	1.00										
BIRTH	0.51	0.63	1.00									
CABG	0.02	−0.04	−0.04	1.00								
CHOL	0.43	0.76	0.72	0.03	1.00							
ENDO	0.40	0.56	0.61	0.08	0.65	1.00						
HAP	0.35	0.51	0.53	0.16	0.57	0.82	1.00					
HIP	0.46	0.74	0.66	0.11	0.78	0.60	0.47	1.00				
KAP	0.36	0.48	0.50	0.15	0.57	0.84	0.83	0.46	1.00			
PNEU	0.43	0.75	0.70	0.06	0.83	0.63	0.54	0.79	0.57	1.00		
STROKE	0.37	0.59	0.59	0.02	0.66	0.60	0.49	0.64	0.51	0.69	1.00	
TOTAL	0.45	0.65	0.76	0.05	0.74	0.73	0.60	0.67	0.60	0.75	0.73	1.00

As the market share MS and the CR3 are reflected in the HHI, it is little surprising that both indicators exhibit a similar pattern as the HHI. Again, even when complex procedures are not considered, the levels of MS and CR3 reached in each of the subsamples is very high and well beyond the thresholds that are used in the German antitrust legislation to indicate highly concentrated markets with potentially negative effects on competition. As expected, the number of competitors NC is inversely related to the concentration indicators. The number of ZIP codes is the only measure that deviates from the familiar pattern between the subsamples. Furthermore, a larger geographic market does not necessarily imply a lower degree of concentration. For a complex procedure, such as a CABG, the relevant geographic market covers a large number of ZIP codes while at the same time the concentration is very high. The average linear distances measured in kilometers that the patients travel to their hospital matches the pattern of the NZIP.

Generally, the subsamples with complex procedures exhibit significantly higher HHI values. Within the standard procedures, there seems to be little difference between surgical and nonsurgical procedures. However, there is interesting variation within the orthopedic surgical procedures. While the HHIs for the subsample on the elective surgeries HAP and KAP is comparatively low (0.19) the HHIs for HIP – which has a very high proportion of emergency admissions – is much higher. While the number of hospital systems is even higher for HIP compared to HAP and KAP – thus prima facie suggesting a similar level of competition – NZIP and the distance indicate that for acute admissions the geographic market is much smaller. These differences vanish once all three subsamples are summarized in the category ORTHO.

**Figure 4:**
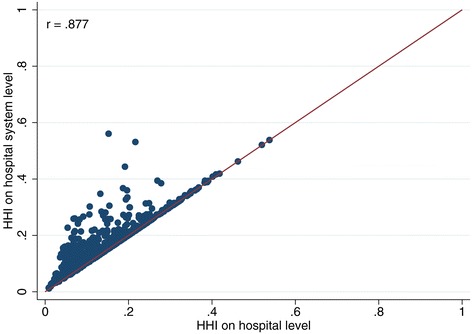
**HHI for subsamples by condition.** Note: PNEU - pneumonia; APP - appendectomy; CHOL - cholecystectomy; HAP - hip arthroplasty; KAP - knee arthroplasty; HIP - hip fracture; ORTHO - joint category for HAP, KAP and HIP; AAA - intact abdominal aortic aneurysm; CABG - coronary artery bypass surgery.

Figure 4 provides a graphical illustration of the distribution of the HHI^HS^ across different procedures. When using the cluster approach for the product market (TOTAL), 799 or 52% of the hospitals are located in markets with a HHI of less than 0.18, i.e. 48% of the hospitals operate in highly concentrated markets. However, Figure 4 also shows a substantial variation in the share of hospitals operating in highly concentrated markets when looking at the single conditions: For AAA and CABG patients, 100% of the hospitals are located in highly concentrated markets, while around 75% (85%) of the hospitals treating patients with the nonsurgical procedures PNEU and STROKE (BIRTH) show higher HHI than the 0.18 threshold. The surgical procedures APP and CHOL are in similar concentrated markets with 75% and 85% of the hospitals, respectively. Slightly less than 50% of the hospitals performing the orthopedic procedures HAP and KAP operate in concentrated markets. For HIP the share increases to 85%, which is comparable to the nonsurgical procedures. When we aggregate all orthopedic procedures together (ORTHO), more than 50% of the hospitals are in concentrated markets. Even HHIs way above 0.6 are a common observation.

The considerable discrepancies between these categories suggest that a highly aggregated cluster market approach disguises severe and systematic differences on a more detailed level. Although it is unlikely that a differentiation on a granular ICD level is practical, more research needs to be dedicated to this aspect. The supply-side substitutability of services captured with ORTHO and the differentiation between elective and acute treatments seem to be promising starting points.

### Geographic market definition

So far, we only considered the 60/01-rule to define the geographic dimension of the hospital market. As pointed out above, there exists no theory based rule to choose the thresholds. Hence, to check for the robustness of our results, we vary the marginal values with 3% and 5% and also consider cumulative thresholds of 40% and 80%.

Table 7 summarizes the results. Starting with the 40/01 rule, increasing the marginal threshold from 1% over 3% to 5% leads to a decrease of the average NZIP from 24.86 to 13.59. This goes along with a reduction of the average NC from 11.06 to 8.01. Inversely, the two concentration measures HHI and CR3 increase from 0.21 to 0.28 and 0.61 to 0.69 respectively. Apparently there are a considerable number of ZIP code areas that contribute between 1% and 3% to hospitals’ patient volume. Considering the cumulative thresholds of 60% and 80%, it is observable that increasing the marginal threshold from 1% to 3% has less effect the higher the cumulative threshold is. This is even more the case for the increase from 3% to 5%. Hence, the cumulative threshold appears to become increasingly binding; to reach the 80% threshold at least in some cases very large numbers of ZIP codes have to be included that account for very small portions, i.e. less than 5% or 3%, of the hospitals case volume.

**Table 7:** **Variation of geographic market thresholds (means)**

**Rule**	**HHI**	**MS**	**CR3**	**NC**	**NZIP**
40/01	0.21	0.29	0.61	11.06	24.86
40/03	0.26	0.36	0.68	8.52	15.35
40/05	0.28	0.38	0.69	8.01	13.59
*60/01* ^*)^	*0.19*	*0.27*	*0.59*	*14.19*	*35.27*
60/03	0.23	0.33	0.64	12.50	28.84
60/05	0.24	0.34	0.65	12.21	27.95
80/01	0.17	0.24	0.55	22.24	64.86
80/03	0.19	0.27	0.57	21.50	62.56
80/05	0.19	0.27	0.57	21.38	62.34

Note: ^*)^ Benchmark scenario.

This becomes even more evident, when the distribution of NC and NZIP is analyzed at in more detail (see Table 8). The mean is mainly driven by a small but still significant share of hospital systems that have a much dispersed constituency. For those, the cumulative threshold is binding and they require excessive numbers of ZIP code areas to reach it. For example, when applying the 80% cumulative threshold more than 5% of all hospitals systems need at least 256 ZIP code areas to reach this figure. A similar pattern can be observed for the number of competitors. The median values for both indicators are also susceptible to a change in the marginal threshold but way less than the mean.

**Table 8:** **Distribution of NC and NZIP under varying thresholds**

**NC**								
**Rule**	**p1**	**p5**	**p25**	**p50**	***mean***	**p75**	**p95**	**p99**
40/01	2	3	6	9	11	14	26	44
40/03	1	1	3	6	9	11	26	39
40/05	1	1	3	5	8	10	26	39
60/01	2	3	6	9	14	17	43	62
60/03	1	2	4	6	12	16	43	62
60/05	1	1	3	6	12	15	43	62
80/01	2	3	6	10	22	24	83	137
80/03	1	2	5	9	21	24	83	137
80/05	1	2	5	9	21	24	83	137
**NZIP**								
**Rule**	**p1**	**p5**	**p25**	**p50**	***mean***	**p75**	**p95**	**p99**
40/01	6	9	17	23	25	29	58	61
40/03	3	5	8	10	15	16	54	61
40/05	2	3	5	7	14	15	54	61
60/01	6	9	17	24	35	37	118	140
60/03	3	5	9	13	29	34	118	140
60/05	2	4	7	13	28	34	118	140
80/01	6	9	18	29	65	74	256	355
80/03	3	6	13	27	63	74	256	355
80/05	3	5	12	27	62	74	256	355

**Figure 5:**
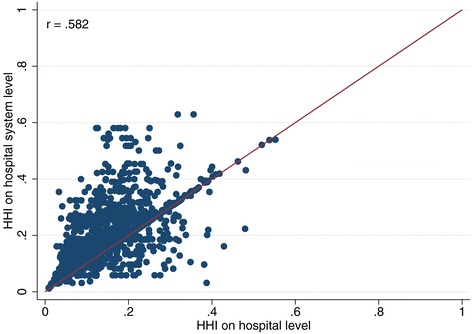
**HHI with varying definitions of the geographic market.**

Again the HHI is much less sensitive to these effects. For example, across all nine rules the 99% percentile of the HHI ranges between 0.54 and 0.67. This is confirmed by Figure 5 in which hospitals are sorted according to their HHI. Obviously, higher cumulative thresholds and lower marginal thresholds tend to result in smaller HHI values. However, contrary to the variations of the product market, the overall picture remains stable. Furthermore, extreme spikes at the upper end of the distribution as have been seen for NC and NZIP cannot be observed.

Calculating the correlation between the HHIs based on the nine different rules supports these findings. For all potential combinations, the correlation ranges between 0.70 and 0.99 (see Table 9).

**Table 9:** **Correlation coefficients of different cumulative-marginal rules**

**Rule**	**40/01**	**40/03**	**40/05**	**60/01**	**60/03**	**60/05**	**80/01**	**80/03**	**80/05**
40/01	1.00								
40/03	0.86	1.00							
40/05	0.80	0.96	1.00						
60/01	0.96	0.87	0.83	1.00					
60/03	0.83	0.93	0.92	0.90	1.00				
60/05	0.78	0.89	0.92	0.86	0.97	1.00			
80/01	0.81	0.76	0.75	0.92	0.89	0.87	1.00		
80/03	0.72	0.76	0.77	0.84	0.91	0.91	0.94	1.00	
80/05	0.70	0.74	0.76	0.83	0.90	0.90	0.93	0.99	1.00

So far, we determined the NC including all hospitals as competitors which treat at least 3% of their patients in a single ZIP code of the relevant market. Table 10 shows how the NC varies if the threshold is modified. With increasing market shares the NC declines. We think that our approach produces a rather conservative figure with a tendency to overestimate the number of relevant competitors. The definition of NC has no effect on the results of HHI, MS and CR3.

**Table 10:** **Number of (significant) competitors for different definitions**

**Definition of significant competitor**	**NC**
3% in a single ZIP-code	14.19
10% in a single ZIP-code	5.87
1% in the whole relevant market	40.54
3% in the whole relevant market	25.61
10% in the whole relevant market	13.25
15% in the whole relevant market	10.43

### Comparison with the benchmark study

We now want to test if the results are robust across datasets. The dataset used by SU is collected and provided by the Federal and State Statistical Offices. The structure is identical to our dataset and it is very likely that the reporting on the hospital level is conducted by the same administrators. However, there is no formal documentation that they actually do use exactly the same data source. As the two datasets cannot be merged, we can only compare descriptive statistics and the final results. The number of hospitals is slightly higher in our dataset (1,517 vs. 1,439), which is mainly due to two reasons: Firstly, while the dataset of the Federal Statistical Office summarizes hospitals with more than one site under one data point, we are able to separate out individual sites. Secondly, in our dataset fewer observations have to be deleted due to missing values. The number of hospital systems is slightly lower in our dataset (910 vs. 944) which is primarily due to a difference regarding the treatment of hospital systems that operate in several states. While SU split hospital systems by states to avoid excessive geographic markets, we refrain from doing so as SU could show that there are no significant differences in concentration measures when testing the two options for robustness. Furthermore, the split of hospital systems along state borders is arbitrary.

For the direct comparison we slightly amend our approach to match exactly the procedure of SU. When calculating the HHI and the CR3 we now only take competitors into account that are deemed relevant as explained in the data and methods section. Doing so, the average HHI calculated on hospital system level is 0.17 which is lower than the HHI of 0.19 in Table 3. SU anticipate this effect of their calculation of the HHI and state that they are likely to underestimate true market concentration.

Exactly replicating the method of SU we obtain the results shown in Table 11. All these results are in line with SU. Only the number of ZIP codes on which the relevant market is constructed differs slightly more. This may be due to the higher number of hospitals in the current sample and the decision to abstain from a state by state split of hospital systems.

**Table 11:** **Concentration measures for the whole hospital sector**

	**N**	**Mean**	**p1**	**p5**	**p25**	**p50**	**p75**	**p95**	**p99**
HHI	1517	0.17	0.01	0.03	0.09	0.14	0.24	0.38	0.54
HHI*	*1384*	*0.17*	*0.01*	*0.02*	*0.09*	*0.14*	*0.23*	*0.40*	*0.61*
MS	1517	0.27	0.00	0.01	0.12	0.26	0.42	0.59	0.73
MS*	*1384*	*0.26*	*0.00*	*0.01*	*0.09*	*0.23*	*0.40*	*0.60*	*0.77*
CR3	1517	0.52	0.14	0.22	0.42	0.52	0.65	0.79	0.84
CR3*	*1384*	*0.54*	*0.11*	*0.19*	*0.42*	*0.56*	*0.67*	*0.80*	*0.88*
NC	1517	14.19	2	3	6	9	17	43	62
NC*	*1384*	*13.39*	*2*	*3*	*6*	*9*	*15*	*37*	*61*
NZIP	1517	35.27	6	9	17	24	37	118	140
NZIP*	*1384*	*25.30*	*3*	*8*	*16*	*23*	*29*	*51*	*79*

Note: *)Benchmark scenario.

## Discussion and conclusion

Looking at the product market definition our findings support the hypothesis that an aggregated cluster market approach neglects relevant information and is a questionable indicator for market concentration. Besides the conceptual argument that it is unlikely that a small local hospital is a relevant competitor across all fields for a neighboring tertiary care provider, our results show that there are considerable differences between different diagnoses. Taking a rigorously static supply side perspective one could argue that strong competition for hip replacement surgery is no alleviation for high concentration and therefore limited choice in the market for cholecystectomies. But even from a perspective that focuses on potential substitutability on the supply side it seems disputable to plainly summarize all diagnoses that might be treated by the same type of specialist within one category. For example, there is a considerable discrepancy concerning the size of the geographic market between elective and emergency admissions. However, the size of the geographic market also varies substantially in-between diagnoses that are characterized by a very low proportion of emergency admissions. Further research is needed to analyze if these differences are driven by the willingness or by the capability of patients to travel for a longer distance to an alternative hospital. If – besides typical emergency admissions – other conditions can be identified that are characterized by a very low capability of patients to travel, this may call for further segmentation of the product market beyond the suggested treatment groups. The very low correlation between the HHIs of the different subsamples and between these and the cluster market HHI underlines that the latter is only of limited use to approximate the market concentration as experienced by the patient. Overall, our results suggest that a generalizing cluster market approach is averaging out many of the severe differences between diagnoses. They further stress the need for additional research of this issue. Empirical literature on this specific topic is scarce, especially as it was usually not the most controversial aspect in merger control cases that were overshadowed by disputes about the delineation of the geographic market. The approach taken by the Office of Fair Trading and the Competition Commission in England [1] seems to be reasonable but was conducted as a comprehensive case study accommodating the very specific situation of the two involved hospitals, thus preventing the generalization of the applied product market differentiation .

In contrast, the analysis of the different cutoff values concerning the definition of the geographic market shows that the results are fairly robust. There are differences regarding the level of concentration, but the correlation is very high and the discrepancies are not as dramatic as for the product market dimension. Again, there is no theoretical basis for an argument in favor of or against one threshold or the other. We argue that the 60/01 rule seems to be a pragmatic compromise. Both the cumulative and the marginal threshold are binding and it is assured that the relevant market reflects more than half of a hospital systems patient volume while not creating excessively large geographic markets for hospital systems with an extremely wide catchment area. As this group seems to be mainly constituted by highly specialized hospitals, this may again interfere with the debate about the correct specification of the product market. Hence, while the correct specification of the geographic market will continue to be a focal controversy in merger control cases, it seems of limited relevance in econometric studies when the HHI (but no other measures like the size of the geographic market) is used as control variable. Nonetheless, depending on the scope of the analysis extensive robustness checks are paramount.

Our results also underline the key findings of the study by SU. We can confirm that the HHI calculated on hospital level is a very poor proxy for true market concentration. A rather low correlation of 0.40 for HHI^H^ and HHI^HS^ questions the econometric validity of such a proxy. Concentration must be calculated on hospital system level. Furthermore, considering the very high level of concentration that is measured across all our different rules and definitions, it seems very reasonable to state that the German hospital market is indeed highly concentrated. While Varkevisser and Schut [28] come to a rather favorable assessment of the rigorousness of the German merger control process – at least compared to the Dutch approach – our results suggest that probably an even stricter course might be advised.

The limited but very consistent evidence on high levels of concentration in the German hospital market puts even more responsibility on policy makers as they may have to reassess some of their positions on the capabilities of selective contracting and the extent to which patients can actually freely choose their hospital.

### Endnotes

^a^There have been fundamental changes in the German hospital sector. In 2004 there was the introduction of a new hospital payment system based on diagnosis related groups (DRG). Prior to this reform, hospital services were mainly reimbursed on a per diem basis. Now payment is linked to cases treated and reflects DRG specific costs averaged across all hospitals within a state. This implies that hospitals have to reduce costs below the average to make profits.

^b^This is because the authority only interacts if the returns of the merging companies exceed a threshold of 500 million Euros which is usually not the case for local and small regional hospital systems.

^c^For a concise review regarding the controversy of the product market definition applied by the German Antitrust Authority see Bangard [19], pp. 214–222, or Jansen [24], pp. 158–201. Commonly, health care is considered to be a differentiated product. This in combination with heterogeneous preferences of patients allows for the emergence of market power in the first place and establishes the need for a deliberate delineation of product markets [13], p. 1411.

^d^They call these groups service categories. Any physician offering treatment within this group could potentially offer treatment for any other treatment within this category. Furthermore, the categories are also differentiated by the qualification level needed to perform these treatments. For example, there is not only a category for General Medicine, but also one for General Medicine Cardiology, indicating that treatments within the latter group require not only knowledge in General Medicine, but further specialization in the field of Cardiology. Sacher and Silvia apply this approach on two regions in California and find that the cluster market approach masks considerable variation on service category level [29].

^e^As Varkevisser et al. point out, the product dimension of the hospital market definition is usually much less contentious. The authors state a “general lack of debate over the relevant product market” ([18], p. 9). Gaynor and Vogt also state the need for more research regarding the definition of the product market for hospital services [13].

^f^The Elzinga-Hogarty approach seeks to identify an area that minimizes exports from and imports into this area below predefined thresholds. It is one of the most common approaches in U.S. hospital merger control investigations and is to some extent reflected by the approach applied by the German Antitrust Authority [7]. However, the method requires a number of case specific ad hoc assumptions to avoid implausible definitions of the geographic market [11,13,22]. Therefore this method is not well suited for the analysis of datasets with large numbers of potential hospital markets.

^g^This does not mean that individual authors did not make statements. For example, Kuchinke and Kallfass argue in favor of the cluster market approach [7] while Jansen states that the cluster market assumption cannot be applied to the hospital market [24]. Coenen et al. remain somewhat undecided [25].

^h^As an example, Dewenter et al. 2013 attempt to measure concentration by the number of beds and disregard hospital system membership. They define circular geographic markets [30]. Other studies face similar problems [31].

^i^For more information on the underlying research project see Schmid [10].

^j^The data was provided by the BKK Federal Association (a health insurance association).

^k^We focus on diagnoses that are closely connected to a procedure as this helps to ensure good data quality. For AAA we use diagnosis and procedure codes defined by the German Federal Joint Committee in their quality assurance agreement for this condition [32]. Diagnosis and procedure codes for patients with hip fracture are taken from the Federal Office for Quality Assurance [33]. For the remaining conditions we use diagnosis and procedure codes on the basis of the German inpatient quality indicators [27]. For a full list of diagnosis and procedure codes used to identify the above mentioned conditions see Table 2.

^l^For a detailed description of the procedure see [10], pp. 110–112.

^m^Up to 2010 the US Horizontal Merger Guidelines suggested the threshold of 0.18 as an indicator for highly concentrated markets. For this reasons, almost all empirical studies refer to this figure. After the revision of the merger guidelines the scale was more differentiated, classifying markets with an HHI between 0.15 and 0.25 as moderately concentrated markets. We stick to the 0.18 indicator as it resembles remarkably well the indicator used in the German merger guidelines (market share of 30% or higher of the merged entity) and allows for a direct comparison with prior studies.

^n^The proxies MS and CR3 are closely related to thresholds established in the German antitrust legislation. §19 GWB states that it can be assumed that a firm has market power if its market share is at least 33% and that a group of three (or less) firms has market power if their accumulated market share reaches 50%.

^o^We also conducted robustness checks with other weights such as the number of patients treated, but the results remained stable.

^p^If looking only at hospitals that belong to a hospital system, the difference increases to 0.091 (N = 834). As expected, with a delta of 0.027 the difference is much smaller for the subsample of stand-alone hospitals (N = 683).

^q^In consequence, when plotting the HHI^H^ and the HHI^HS^ only for stand-alone hospitals in a scatter plot, the dots are clustered very closely along the diagonal.
